# GFS: fuzzy preprocessing for effective gene expression analysis

**DOI:** 10.1186/s12859-016-1327-8

**Published:** 2016-12-23

**Authors:** Abha Belorkar, Limsoon Wong

**Affiliations:** School of Computing, National University of Singapore, 13 Computing Drive, Singapore, 117417 Republic of Singapore

**Keywords:** Gene expression analysis, Fuzzy scoring, Preprocessing, Normalization

## Abstract

**Background:**

Gene expression data produced on high-throughput platforms such as microarrays is susceptible to much variation that obscures useful biological information. Therefore, preprocessing data with a suitable normalization method is necessary, and has a direct and massive impact on the quality of downstream data analysis. However, it is known that standard normalization methods perform poorly, specially in the presence of substantial batch effects and heterogeneity in gene expression data.

**Results:**

We present Gene Fuzzy Score (GFS), a simple preprocessing technique, that is able to largely reduce obscuring variation while retaining useful biological information. Using four sets of publicly available datasets containing batch effects and heterogeneity, we compare GFS with three standard normalization techniques as well as raw gene expression. Each method is evaluated with respect to the quality, consistency, and biological coherence of its processed output. It is found that GFS outperforms other transformation techniques in all three aspects.

**Conclusion:**

Our approach to preprocessing is a stronger alternative to popular normalization techniques. We demonstrate that it achieves the essential goal of preprocessing – it is effective at making expression values from multiple samples comparable, even when they are from separate platforms, in independent batches, or belong to a heterogeneous phenotype.

## Introduction

Gene expression profiling experiments and analysis are often designed with the objective of verifying one or more hypotheses that can help in building effective diagnostic or prognostic models in clinical settings. Typically, expression data are collected from groups manifesting differences in certain properties of interest, such as disease types or states, developmental stages, and response to specific treatments or interventions over time. The collected data are then mined for appropriate variation patterns relevant to the hypotheses under consideration. The underlying assumption in such studies is that the input gene expression values from different samples accurately reflect the amounts of RNA produced by the corresponding genes and, thus, are properly comparable. However, in practice, unless an effective normalization technique is applied to preprocess the expression data, a number of factors may lead to the violation of this assumption [1, 2].

Firstly, the entire technical process of isolation and quantification of RNA leading up to the final measurements is unlikely to be completely error-free, as inaccuracies may insinuate any of the steps in the long procedure. Secondly, with change in time, place, and other variables in experimental settings, systematic biases of non-biological origins invariably enter during measurement experiments in the form of batch effects. When such biases are correlated with the biological properties under investigation, they can severely confound interesting variation [3]. Thirdly, differences in experimental settings may also introduce changes in local environments of cells, thus inducing fluctuations in gene expression that further contribute to noise in the measurement data [4].

All these factors together make it improbable for multiple samples to naturally have comparable expression values. Therefore, we rely heavily on the capabilities of a preprocessing method to recover meaningful biological information, and remove or account for noise in the form of obscuring variation. Yet, it was reported [2] that popular normalization techniques are not very successful in discriminating between real and obscuring variation to produce quality input for downstream gene expression analysis. In fact, it was noted by Luo et al. [2] that preprocessing using common methods led to reduction in the quality of subsequent predictive models in upto 25% of the cases.

To mitigate the performance issues commonly presented by preprocessing techniques, we propose Gene Fuzzy Score (GFS), a transformation method that uses fuzzy scores derived from rank values of gene expression within individual samples. We chose four different sets of gene expression data containing substantial batch effects and heterogeneity for the analysis. On these datasets, we compared the performance of GFS and other popular preprocessing methods with respect to the quality, consistency, and biological coherence of their processed output.

## Background

Preprocessing techniques typically attempt to make expression values from multiple samples comparable in two different ways:

1. by scaling expression values such that each sample has an equal value for a statistic such as mean or median; or
2. by adjusting expression values such that each sample has the same expression distribution across genes.

The first approach includes methods such as mean and median scaling, and is popular for Affymetrix genechips. For example, in the mean scaling method, the mean gene expression value of each microarray in the sample is first calculated, and a grand mean is then computed as the mean of all means. Finally, expression value of each microarray in the sample is scaled such that the mean expression of each microarray is equal to the grand mean. Median scaling also follows the same procedure, with the mean statistic being replaced by median. While these methods are simple to implement, they assume that expression values of all samples share a linear relationship. They – especially mean scaling – also suffer from a few other drawbacks such as sensitivity to outlier distortions [5].

The second approach includes more sophisticated methods such as z-score and quantile normalization. In z-score normalization, the expression values of genes in each microarray are transformed to fit the standard normal distribution with a mean of zero and 1 unit standard deviation. On the other hand, quantile normalization uses the rank values of gene expression within individual microarrays to make the distribution of all microarrays identical in statistical properties. Since ranks are known to be relatively more robust to batch effects than absolute expression values [1], this is expected to lead to better performance on datasets with batch effects. In the quantile normalization procedure, the expression values of each microarray are first sorted in ascending order, and the mean expression corresponding to each rank across microarrays is stored separately. Following this, the original expression values in each microarray are assigned ranks based on their relative quantitative order. Finally, a transformed matrix is obtained by replacing each gene rank value by the mean expression value corresponding to that rank as stored earlier.

The z-score and quantile normalization methods are relatively more robust to outliers, provided that the number of microarrays in a dataset is sufficiently large. However, the actual distributions of underlying data are assumed to be identical in all samples, and specifically assumed to be Gaussian in case of z-score normalization. This assumption is especially likely to break down in datasets with disease state samples where the regular functions of the genes and their synchronization with each other may be substantially disrupted. In such cases, the expression patterns within a disease sample may not be identical to samples of the normal phenotype. It also may not be identical to other disease samples if the disease is heterogeneous and is able to manifest itself through the exploitation and/or breaking of multiple mechanisms.

It is also commonly observed that low-expression genes and proteins exhibit a much greater coefficient of variance than highly expressed ones in their expression levels (see figure 2E in the work by Goh et al. [6]). Thus, the expression rank of low-expression genes is highly unstable. This may adversely affect the performance of a ranking-based normalization method such as quantile normalization.

Therefore, we are inspired to present GFS as a preprocessing technique for gene expression. Like quantile normalization, our method also makes use of gene expression ranks instead of absolute values, thus earning more robustness to batch effects. However, unlike the above techniques, we do not make any assumptions on the similarity of distribution or the equality of any mean-, median-like statistic across samples. Moreover, in our method, we fuzzify the expression ranks such that irrelevant fluctuations introduced by minor differences in ranks are alleviated, and noise from low-ranked genes is discarded.

The idea of fuzzification has also been used earlier in a few gene expression profile analysis methods [7, 8] and also proteomic profile analysis methods [6, 9]. However, these works merely use it as a component of their respective methods, and do not study its role and effectiveness as a normalization procedure.

## Methods

### Datasets

We collected datasets (see Table 1) from three different disease types – Duschenne Muscular Dystrophy (DMD), Leukemia, and Acute Lymphoblastic Leukemia (ALL).

**Table 1:** Datasets used for comparing preprocessing methods

Disease type	Source	Affy GeneChip	Dataset composition
DMD	Haslett et al. [10]	HG-U95Av2	12 DMD, 12 controls
	Pescatori et al. [11]	HG-U133A	22 DMD, 14 controls
Leukemia	Golub et al. [13]	HU-6800	47 ALL, 25 AML
	Armstrong et al. [12]	HG-U95Av2	24 ALL, 24 AML
ALL	Yeoh et al. [14]	HG-U95Av2	15 BCR-ABL, 27 E2A-PBX1
	Ross et al. [15]	HG-U133A	15 BCR-ABL, 18 E2A-PBX1
ALL	Yeoh et al. [14]	HG-U95Av2	6 Normal, 26 TEL-AML1,
			22 Hyperdip >50, 15 T-ALL,
			10 Pseudodip, 6 BCR-ABL,
			7 MLL, 8 Hyperdip47-50
			9 E2A-PBX1, 3 Hypodip

A single gene expression matrix was produced by merging the two DMD datasets from Haslett et al. [10] and Pescatori et al. [11]. Similarly, data were merged from Armstrong et al. [12] and Golub et al. (Leukemia) [13], as also from Yeoh et al. [14] and Ross et al. (ALL subtypes) [15].

Note that each of the first three pairs of the chosen datasets (as in Table 1) are independent and were produced on different microarray platforms. Thus, the merged gene expression matrices obtained from them contain batch effects by default. We consider only genes that are common in the two samples of the dataset pair, and run all the four preprocessing techniques – GFS, mean scaling, z-score normalization, and quantile normalization – on these input matrices, and evaluate their effectiveness in dealing with batch effects. To observe the effect of preprocessing on highly heterogenous data, we also use another more heterogeneous dataset from Yeoh et al. [14] that has 9 disease subtypes (ALL) and normal patient samples to compare the selected methods. Thus, in total, four sets of input gene expression matrices belonging to three different disease types are used in our analysis.

### Approach

In GFS, we transform a raw gene expression matrix by making use of the rank values of genes within each microarray, rather than by using their absolute expression values. Further, we use two quantile thresholds – *θ*_1_ and *θ*_2_ – to assign a fuzzified score to each gene in each patient. Ranks below *θ*_2_ in a sample are all reduced to a score of zero, those above *θ*_1_ are given a score of 1, and intermediate ranks are interpolated to obtain a score between 0 and 1. In particular, let *r*(*g*_*i*_,*p*_*j*_) be the rank of gene expression of a gene *g*_*i*_ in patient *p*_*j*_, and *q*(*p*_*j*_,*θ*) be the rank corresponding to the upper *θ*th quantile of gene expression in patient *p*_*j*_. Then, the gene fuzzy score *s*(*g*_*i*_,*p*_*j*_) assigned to a gene *g*_*i*_ in patient *p*_*j*_ is given by the following function:

1 $${} s(g_{i}, p_{j})= \left\{\!\!\begin{array}{lll} 1, & \text{if}~q(p_{j}, \theta_{1}) < r(g_{i}, p_{j}) \\ \frac{r(g_{i}, p_{j})-q(p_{j}, \theta_{2})}{q(p_{j}, \theta_{1})-q(p_{j}, \theta_{2})},\!\!\!& \text{if}~q(p_{j}, \theta_{1}) > r(g_{i}, p_{j}) \geq q(p_{j}, \theta_{2}\!)\\ 0, & \text{otherwise} \end{array}\right.  $$

Apart from the use of rank values in computing transformed scores, GFS also benefits from the fact that it allows for selection of quantile thresholds such that noise from low-ranked genes is safely removed by assigning a score of 0, while genes with very high expression are all treated equally with a score of 1. For the purpose of uniformity in comparison, we fix *θ*_1_ to 5% and *θ*_2_ to 15% for all GFS runs mentioned in this paper. However, using a *θ*_1_ value between 5 to 10% and *θ*_2_ value between 15 to 20% also leads to similar results.

In evaluating the proposed approach against other normalization techniques discussed earlier, we focus on three salient questions in this paper:

1. Does the preprocessing technique produce consistent results across different datasets, provided that they have the same composition of different phenotypes?
2. What is the quality of the output produced by the processing technique? How well does the processing retain useful information while mitigating obscuring effects?
3. Is the output produced by the technique biologically coherent?

We compared GFS with three standard normalization methods described in the previous section – mean scaling, z-score normalization, and quantile normalization. The description of our design and approach to each experiment is given in the next section.

## Results and Discussion

### Visualizing data after PCA transformation

We preprocess the raw gene expression matrices with each of the four methods – mean scaling, z-score normalization, quantile normalization and GFS. For each method, we select the top 15% genes with maximum variance in the processed matrix, as these are most likely to be the genes contributing to interesting variation. We then reduce the processed matrix to include only these high variance genes, and apply PCA transformation on the reduced matrix. A scatter plot of the coordinates corresponding to the first two principal components (PC1 and PC2) of each sample is visualized.

A good preprocessing method is expected to show a clear clustering of samples of the same phenotype, and separation between samples of different phenotypes. Moreover, the quality of clustering would ideally not be adversely affected by the presence of samples from multiple batches in the data.

**Fig. 1:**
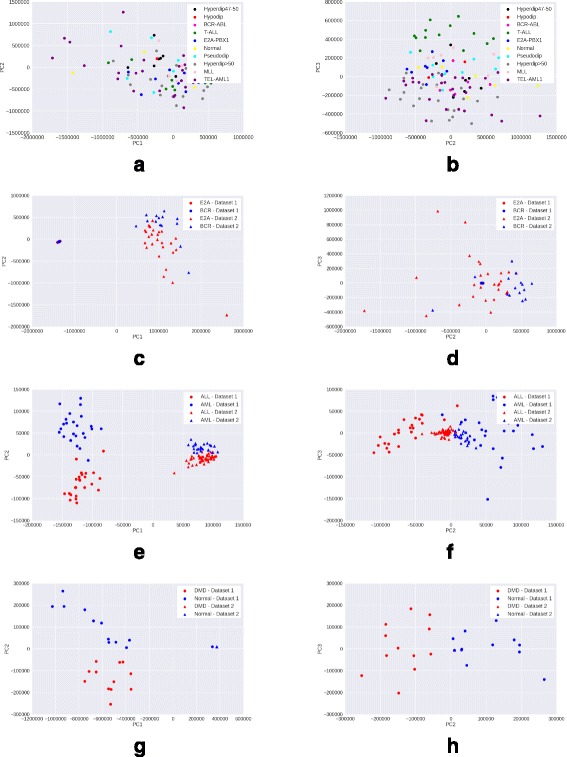
Visualisation with PCA scatter plots – Raw expression. **a** ALL (9 subtypes): PC1 vs. PC2. **b** ALL (9 Subtypes): PC2 vs. PC3. **c** ALL (2 subtypes): PC1 vs. PC2. **d** ALL (2 Subtypes): PC2 vs. PC3. **e** Leukemia : PC1 vs. PC2. **f** Leukemia : PC2 vs. PC3. **g** DMD: PC1 vs. PC2. **h** DMD: PC2 vs. PC3

**Fig. 2:**
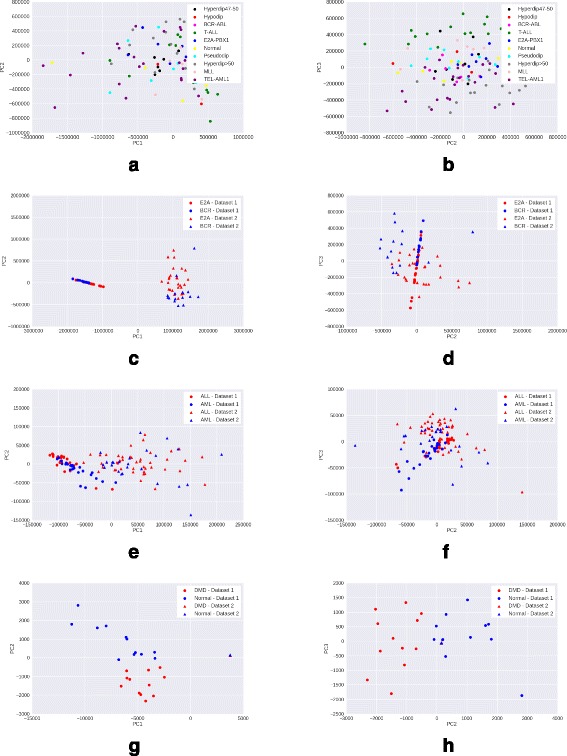
Visualisation with PCA scatter plots – Mean-scaled expression. **a** ALL (9 subtypes): PC1 vs. PC2. **b** ALL (9 Subtypes): PC2 vs. PC3. **c** ALL (2 subtypes): PC1 vs. PC2. **d** ALL (2 Subtypes): PC2 vs. PC3. **e** Leukemia: PC1 vs. PC2. **f** Leukemia: PC2 vs. PC3. **g** DMD: PC vs. PC2. **h** DMD: PC2 vs. PC3

**Fig. 3:**
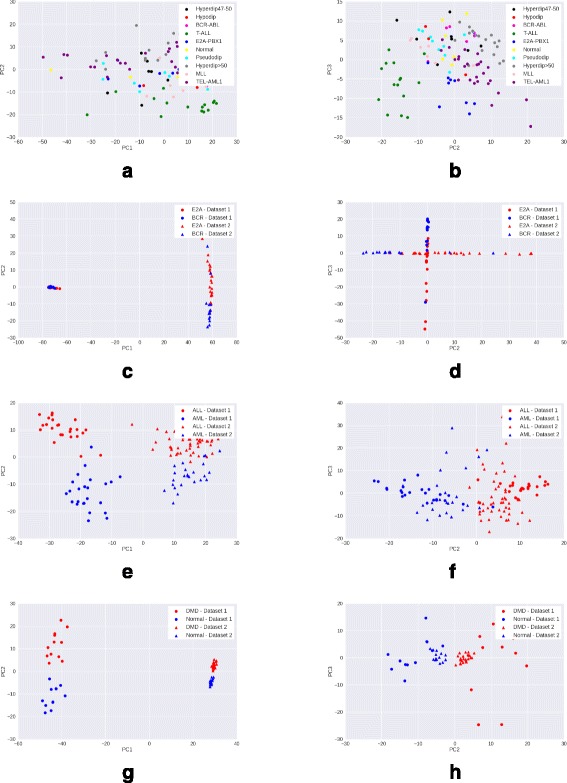
Visualisation with PCA scatter plots – Z-score normalized expression. **a** ALL (9 subtypes): PC1 vs PC2. **b** ALL (9 Subtypes): PC2 vs. PC3. **c** ALL (2 subtypes): PC1 vs PC2. **d** ALL (2 Subtypes): PC2 vs. PC3. **e** Leukemia: PC1 vs. PC2. **f** Leukemia: PC2 vs. PC3. **g** DMD: PC1 vs. PC2. **h** DMD: PC2 vs. PC3

**Fig. 4:**
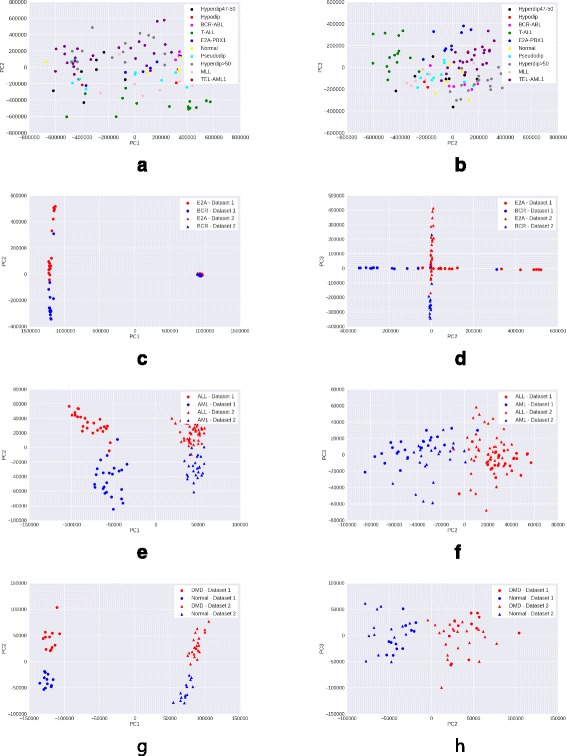
Visualisation with PCA scatter plots – Quantile normalized expression. **a** ALL (9 subtypes): PC1 vs. PC2. **b** ALL (9 Subtypes): PC2 vs. PC3. **c** ALL (2 subtypes): PC1 vs. PC2. **d** ALL (2 subtypes): PC2 vs. PC3. **e** Leukemia: Pc1 vs. PC2. **f** Leukemia: PC2 vs. PC3. **g** DMD: PC1 vs. PC2. **h** DMD: PC2 vs. PC3

**Fig. 5:**
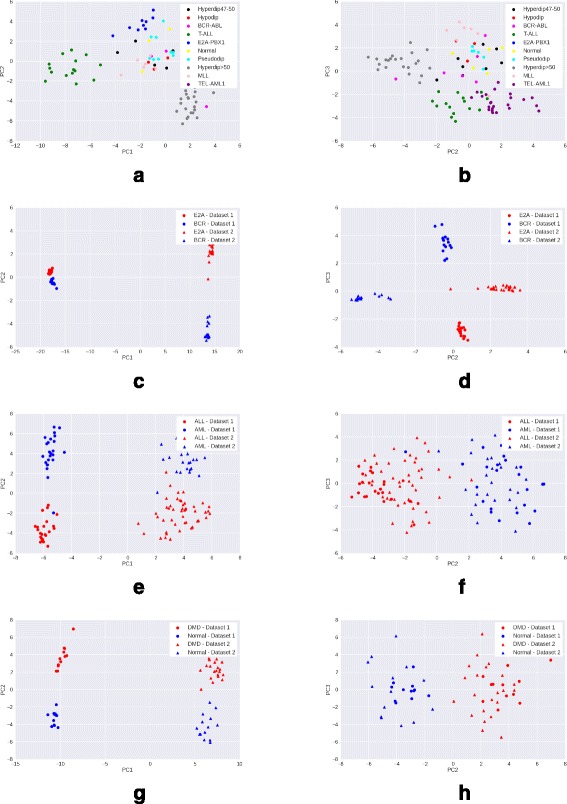
Visualisation with PCA scatter plots – GFS normalized expression. **a** ALL (9 subtypes): PC1 vs. PC2. **b** ALL (9 Subtypes): PC2 vs. PC3. **c** ALL (2 subtypes): PC1 vs. PC2. **d** ALL (2 Subtypes): PC2 vs. PC3. **e** Leukemia: PC1 vs. PC2. **f** Leukemia: PC2 vs. PC3. **g** DMD: PC1 vs. PC2. **h** DMD: PC vs. PC3

*Observations*: While in the Leukemia, DMD, and childhood ALL datasets, samples from different batches are clearly separated, GFS (Fig. 5) shows the best phenotype-wise clustering of samples among all preprocessing techniques. Mean scaling (Fig. 2) does not perform well on any of the datasets, and in some cases, obscures the separation seen even in raw gene expression (Fig. 1). This degredation in peformance is in line with previous findings [2]. Z-score normalization shows good performance on DMD and Leukemia (Fig. 3) datasets, and quantile normalization performs well only on the DMD dataset (Fig. 4).

In case of the more heterogeneous ALL dataset (9 disease subtypes and normal samples), GFS is the only method to discriminate between samples of the different ALL subtypes (Figs. 1, 2, 3, 4, 5 (a)).

From the PCA scatterplots for all the three datasets with batch effects (Leukemia, DMD, and ALL with 2 subtypes), we observed that samples from two batches are always clearly separated along PC1. This implies that the first principal component is highly enriched in batch effects. Therefore, we exclude the first principal component (PC1), and draw scatterplots corresponding to the second and third principal component (PC2, PC3). In PC2 vs PC3 scatterplots, there is very less separation between samples from different batches but belonging to the same phenotype, as compared to that in PC1 vs PC2 scatterplots (Figs. 1, 2, 3, 4, 5). This trend is consistent across all three datasets with batch effects. Thus, removing PC1 can be an effective technique to reduce batch effects in gene expression data to a great extent. However, for the more heterogeneous ALL dataset where batch effects are absent, removing PC1 results in loss of important variation information, and subsequently, less clearer separation between different phenotypes.

### Comparing processing quality

Quality of a preprocessing method is determined by its ability to separate interesting from obscuring variation. An inferior preprocessing method will lead to an output in which expression variation across microarrays would be confounded with irrelevant information. In contrast, expression variation across microarrays in the output of an ideal preprocessing method will correspond to interesting biological variation alone.

*Experiment*: We estimate the quality of preprocessing methods with respect to the capability of their transformed output to separate samples of different phenotypes. In particular, we randomly select 15% of the genes, reduce the processed matrix to include the selected genes, and apply PCA on the resultant matrix. The PCA co-ordinates of all samples are then used to compute a clustering performance metric called the silhouette score. The silhouette score is calculated based on the mean intra-cluster distance *a* and the mean nearest-cluster distance *b* for each sample, as (*b*−*a*)/*m* *a* *x*(*a*,*b*) [16]. The score ranges from -1 to 1. In general, a higher silhouette score indicates a better clustering.

For the ALL dataset with 9 subtypes, co-ordinates corresponding to the first three principal components are used, while for the other three datasets with batch effects, co-ordinates corresponding to only the second and third principal components are used. This is repeated over 1000 iterations, and the distribution of silhouette scores corresponding to each preprocessing method is used to infer the quality of clusters formed by its transformed output.

**Fig. 6:**
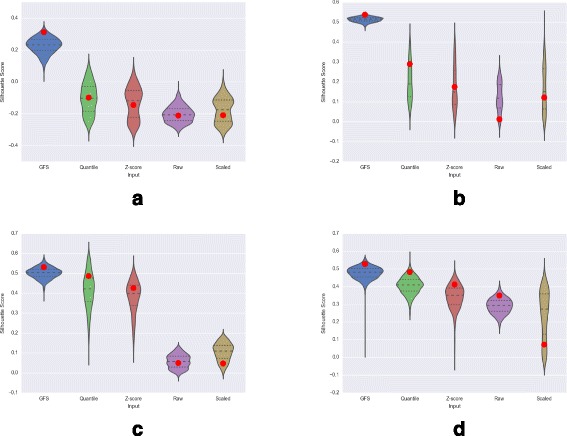
Null distributions of silhouette scores obtained with raw and processed expression matrices taking 15% random genes as features (the *three dashed lines* show 25th quartile, median and 75th quartile, while the *red dot* indicates the score obtained from top 15% variance genes). **a** ALL (9 Subtypes). **b** ALL (2 Subtypes). **c** DMD. **d** Leukemia

*Observations*: For all the four datasets, the distribution of silhouette scores obtained using randomly chosen 15% genes is stable at a higher value in case of GFS, in comparison to other preprocessing methods (see Fig. 6). This shows that the assigned scores to each microarray-gene pair after GFS preprocessing are more relevant to the interesting variation in gene expression and thus, even randomly chosen features are better able to capture the phenotype-based clusters. Moreover, the reference silhouette scores obtained from the top 15% variance genes in GFS processed matrices are consistently higher than the 75th percentile score of its null distribution obtained from random 15% genes, across all datasets (Fig. 6). For quantile normalization, while the silhouette scores obtained from its top 15% variance genes are also consistently higher than the 75th percentile score of the corresponding null distribution, these observed silhouette scores are consistently lower than those for GFS. On the other hand, the silhouette scores derived using the top 15% variance genes in z-score normalized and raw expression are lower than the 75th percentile score of their corresponding null distributions in the DMD dataset and ALL dataset with 2 subtypes. The silhouette score computed on top 15% variance genes in scaled expression data is lower than the median score of its null distribution in all datasets. This shows GFS-processed expression values are more effective than the other methods.

The silhouette scores obtained from the PCA transformed co-ordinates of samples using the top 15% high-variance genes are recorded in Tables 2 and 3. In all datasets, with and without the first principal component (which is often the richest in batch effects), GFS is seen to have a better score relative to other processing methods. Also, in the three datasets with batch effects, removing PC1 improves phenotype-wise clustering, while in the heterogeneous ALL dataset with no batch effects, removing PC1 leads to discarding important variation and thus a reduction in clustering performance.

**Table 2:** Silhouette Scores obtained using the transformed expression values from top 15% variance genes on applying different preprocessing techniques (using first three principal components)

	Raw	Scaled	Z-Score	Quantile	GFS
ALL (9 subtypes)	-0.212	-0.209	-0.145	-0.099	**0.312**
ALL (2 subtypes)	0.009	0.027	0.043	0.070	**0.145**
DMD	0.025	0.044	0.096	0.202	**0.203**
Leukemia	0.153	0.128	0.177	0.227	**0.289**

Silhouette scores corresponding to GFS are the highest among all methods (highlighted in bold)

**Table 3:** Silhouette Scores obtained using the transformed expression values from top 15% variance genes on applying different preprocessing techniques (using only PC2 and PC3, ignoring PC1)

	Raw	Scaled	Z-Score	Quantile	GFS
ALL (9 subtypes)	-0.243	-0.186	0.017	0.027	**0.217**
ALL (2 subtypes)	0.012	0.121	0.176	0.289	**0.538**
DMD	0.049	0.047	0.426	0.486	**0.530**
Leukemia	0.349	0.072	0.412	0.482	**0.528**

Silhouette scores corresponding to GFS are the highest among all methods (highlighted in bold)

### Comparing consistency

It is important that a reliable preprocessing method produces an output that remains consistent in multiple runs over datasets of the same type. For instance, if two datasets of the same disease are transformed by a preprocessing method, and the genes indicated to have the highest contribution to interesting variation have very little overlap, it is natural to infer that the variation is confounded by noise and the genes are likely to be false positives. In contrast, consistency in such output affirms that the preprocessing method is indeed reliable, since similarity in input ensures similarity in output. Thus, a preprocessing technique assigning meaningfully transformed expression values should indicate a consistent set of high-variance genes, when applied to different datasets with the same phenotype distribution.

*Experiment*: In order to evaluate the consistency of different preprocessing methods, we split each dataset into two datasets such that each contains the same number of samples of each phenotype, independently apply the preprocessing technique on the resultant split data, and obtain the two resulting lists of the top 15% high-variance genes from the splits. Further, we apply PCA to the normalized data, and remove genes that have a coefficient of zero in all of the first three principal components for the ALL dataset with 9 disease subtypes. For the other three batch effects-ridden datasets, we only remove genes that have a coefficient of zero in the second and third principal component. This process is repeated 100 times using different splits of each dataset. We then examine the distribution of similarity (measured in terms of the jaccard coefficient) between the two gene lists.

**Fig. 7:**
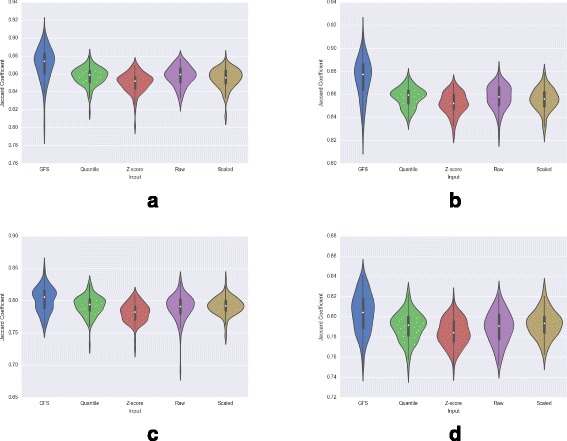
Consistency of preprocessed output - Jaccard coefficient distribution of *top* variance contributing genes on comparing 100 data splits. **a** ALL (9 Subtypes). **b** ALL (2 Subtypes). **c** DMD. **d** Leukemia

*Observations*: A consistent preprocessing technique would be expected to demonstrate a high overlap in high-variance genes. It is seen that the distribution of jaccard coefficient when the split datasets are processed using GFS, is stable at an equal or higher value in all the datasets (Fig. 7). The other methods fluctuate in performance and, in some cases, show worse consistency than raw gene expression.

### Comparing biological coherence

For a phenotype to manifest, the causal genes often co-ordinate with other genes, and seldom act alone. Therefore, genes contributing to interesting variation in data are more likely to be connected to each other in biological pathways. Thus, we expect that a more biologically coherent preprocessing technique will result in high-variance genes that induce significantly more and/or bigger subnetworks on known biological pathways.

*Experiment*: We assess the biological coherence of the preprocessing methods by examining the subnetwork size distribution obtained when high-variance genes are used to induce subnetworks on pathways. The subnetwork size distribution for each processing method is obtained as follows:

1. Preprocess the gene expression matrix using the chosen technique.
2. Select top 15% genes with maximum variance across patient samples.
3. Reduce processed expression matrices to only include the selected genes.
4. Perform a PCA transformation on the reduced matrix, and list genes with non-zero coefficients in any of the first three principal components.
5. Using genes in step 4, induce subnetworks on known pathways from the PathwayAPI database [17] and store the subnetwork size distribution.

To generate the null model, step 2 is replaced with randomly selecting 15% of all genes, and steps 1–5 are repeated over 1000 iterations. Finally, for each subnetwork size, a *p*-value is calculated as the proportion of subnetwork frequencies in the null model found to be greater than the frequency from original distribution.

The same analysis is repeated for the three datasets with batch effects by modifying step 4 to include only those genes that have a non-zero coefficient in the second or third principal component.

**Fig. 8:**
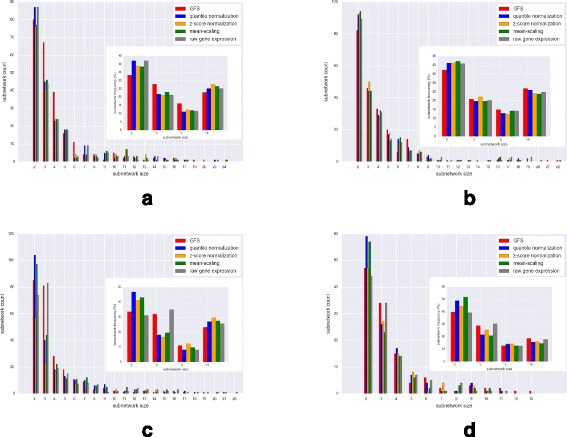
Distribution for size of subnetworks induced by high-variance genes in different preprocessed outputs (using first three components); Inset figure shows the same as percentage frequency. **a** ALL (9 Subtypes). **b** ALL (2 Subtypes). **c** DMD. **d** Leukemia

**Fig. 9:**
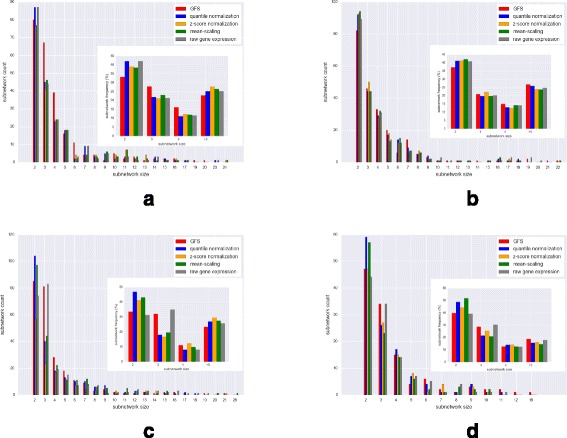
Distribution for size of subnetworks induced by high-variance genes in different preprocessed outputs (using PC2, PC3 only, ignoring PC1 from analysis); Inset figure shows the same as percentage frequency. **a** ALL (9 Subtypes). **b** ALL (2 Subtypes). **c** DMD. **d** Leukemia

*Observations*: The distribution of subnetwork sizes induced by the top 15% variance genes are shown in Fig. 8 (using the first three principal components) and Fig. 9 (using PC2 and PC3 only). The figures show the actual subnetwork count distribution across different subnetwork sizes, while the inset figures show the corresponding percentage frequencies. In the Leukemia dataset and ALL dataset with 2 subtypes, GFS has the highest percentage frequency of subnetworks of size greater than or equal to 5 and, in most datasets, GFS induces more subnetworks overall.

From the low *p*-values in Tables 4, 5, 6, 7, we observe that the significance of frequencies is high for subnetworks induced by GFS, regardless of their size. Further, comparison with other methods shows that the frequency of subnetworks induced by high-variance genes in GFS-processed datasets is much more significant than those induced on datasets processed with other methods and raw gene expression. Hence, we infer that GFS-transformed output is highly biologically coherent. Moreover, we observe that on excluding the batch effects-enriched PC1 from the analysis, the *p*-values corresponding to larger subnetwork sizes are lower than those of smaller sizes, indicating higher significance, and hence greater biological coherence, of the large subnetwork sizes.

**Table 4:** Leukemia – Significance comparison of size of subnetworks induced by high-variance genes in preprocessed output; *p*_1_ = *p*-value using first three PCs, *p*_2_ = *p*-value using PC2, PC3 only

	Raw			Scaled			Z-score			Quantile			GFS		
Size	freq	*p*_1_	*p*_2_	freq	*p*_1_	*p*_2_	freq	*p*_1_	*p*_2_	freq	*p*_1_	*p*_2_	freq	*p*_1_	*p*_2_
2	44	0.994	0.993	57	0.920	0.924	47	0.987	0.986	59	0.893	0.894	47	0.570	0.582
3	34	0.557	0.584	23	0.954	0.945	27	0.842	0.844	26	0.885	0.883	34	0.073	0.075
4	14	0.664	0.679	14	0.664	0.679	15	0.588	0.589	17	0.454	0.471	15	0.046	0.044
5	7	0.597	0.579	6	0.700	0.686	8	0.474	0.465	7	0.597	0.579	4	0.244	0.253
6	5	0.279	0.318	2	0.762	0.779	1	0.904	0.925	4	0.423	0.462	6	0.011	0.013
7	1	0.688	0.696	1	0.688	0.696	4	0.130	0.149	1	0.688	0.696	2	0.159	0.166
8	4	0.048	0.039	3	0.119	0.104	-	-	-	1	0.487	0.500	1	0.259	0.220
9	1	0.384	0.369	2	0.153	0.159	3	0.051	0.047	4	0.014	0.011	3	0.021	0.017
10	1	0.285	0.252	2	0.107	0.098	1	0.285	0.252	1	0.285	0.252	2	0.032	0.031
11	1	0.201	0.224	-	-	-	-	-	-	1	0.201	0.224	2	0.020	0.017
12	-	-	-	-	-	-	-	-	-	-	-	-	1	0.030	0.028
15	-	-	-	-	-	-	-	-	-	-	-	-	1	0.006	0.001

**Table 5:** ALL (2 Subtypes) – Significance comparison of size of subnetworks induced by high-variance genes in preprocessed output; *p*_1_ = *p*-value using first three PCs, *p*_2_ = *p*-value using PC2, PC3 only

	Raw			Scaled			Z-score			Quantile			GFS		
Size	freq	*p*_1_	*p*_2_	freq	*p*_1_	*p*_2_	freq	*p*_1_	*p*_2_	freq	*p*_1_	*p*_2_	freq	*p*_1_	*p*_2_
2	89	0.620	0.604	94	0.476	0.482	93	0.502	0.509	92	0.527	0.532	82	0.128	0.105
3	44	0.646	0.663	44	0.646	0.663	50	0.419	0.430	44	0.646	0.663	46	0.030	0.030
4	31	0.196	0.173	32	0.162	0.153	28	0.312	0.316	29	0.268	0.259	33	0.001	0.001
5	14	0.429	0.398	13	0.509	0.487	18	0.169	0.156	17	0.226	0.193	20	0.001	0.002
6	12	0.082	0.101	15	0.018	0.024	12	0.082	0.101	14	0.032	0.038	6	0.045	0.043
7	7	0.133	0.117	7	0.133	0.117	6	0.224	0.220	9	0.035	0.030	14	0.000	0.000
8	6	0.050	0.043	6	0.050	0.043	7	0.019	0.017	5	0.098	0.097	5	0.006	0.005
9	2	0.324	0.345	2	0.324	0.345	1	0.594	0.607	4	0.061	0.069	3	0.043	0.031
10	3	0.076	0.075	1	0.451	0.449	1	0.451	0.449	1	0.451	0.449	1	0.177	0.168
11	1	0.350	0.357	-	-	-	-	-	-	-	-	-	1	0.129	0.117
12	1	0.300	0.278	1	0.300	0.278	1	0.300	0.278	1	0.300	0.278	1	0.066	0.083
13	-	-	-	1	0.233	0.264	1	0.233	0.264	1	0.233	0.264	-	-	-
14	-	-	-	-	-	-	-	-	-	-	-	-	1	0.021	0.019
15	-	-	-	1	0.156	0.145	1	0.156	0.145	1	0.156	0.145	-	-	-
16	1	0.133	0.139	3	0.005	0.002	2	0.038	0.027	2	0.038	0.027	1	0.002	0.005
17	3	0.006	0.001	1	0.093	0.099	2	0.020	0.018	1	0.093	0.099	1	0.003	0.002
18	-	-	-	1	0.077	0.070	1	0.077	0.070	2	0.013	0.012	1	0.000	0.001
19	3	0.001	0.001	-	-	-	1	0.008	0.007	-	-	-	2	0.000	0.000
20	1	0.035	0.041	-	-	-	-	-	-	-	-	-	-	-	-
21	-	-	-	-	-	-	-	-	-	-	-	-	1	0.000	0.000
22	-	-	-	1	0.008	0.007	-	-	-	-	-	-	1	0.000	0.000

**Table 6:** DMD – Significance comparison of size of subnetworks induced by high-variance genes in preprocessed output; *p*_1_ = *p*-value using first three PCs, *p*_2_ = *p*-value using PC2, PC3 only

	Raw			Scaled			Z-score			Quantile			GFS		
Size	freq	*p*_1_	*p*_2_	freq	*p*_1_	*p*_2_	freq	*p*_1_	*p*_2_	freq	*p*_1_	*p*_2_	freq	*p*_1_	*p*_2_
2	74	0.901	0.903	970	0.429	0.415	57	0.995	0.995	104	0.298	0.278	85	0.015	0.009
3	83	0.004	0.007	44	0.649	0.644	23	0.999	0.999	40	0.794	0.777	81	0.000	0.000
4	19	0.817	0.799	22	0.660	0.643	17	0.894	0.894	18	0.861	0.861	28	0.002	0.004
5	15	0.337	0.324	11	0.692	0.665	12	0.588	0.586	13	0.499	0.485	18	0.001	0.000
6	7	0.536	0.521	11	0.147	0.145	7	0.536	0.521	10	0.213	0.206	11	0.000	0.000
7	8	0.084	0.106	12	0.005	0.005	4	0.521	0.519	10	0.021	0.022	9	0.000	0.000
8	7	0.025	0.018	6	0.053	0.045	3	0.379	0.392	6	0.053	0.045	3	0.019	0.011
9	1	0.598	0.615	5	0.029	0.031	3	0.182	0.148	7	0.004	0.008	4	0.000	0.002
10	2	0.209	0.229	1	0.449	0.467	3	0.089	0.084	2	0.209	0.229	2	0.007	0.007
11	2	0.134	0.140	5	0.001	0.001	1	0.372	0.372	2	0.134	0.140	1	0.012	0.006
12	4	0.006	0.003	3	0.021	0.027	-	-	-	3	0.021	0.027	1	0.005	0.003
13	3	0.017	0.016	2	0.078	0.077	3	0.017	0.016	2	0.078	0.077	2	0.000	0.001
14	3	0.011	0.012	1	0.200	0.189	3	0.011	0.012	-	-	-	1	0.000	0.002
15	2	0.054	0.039	3	0.012	0.009	1	0.181	0.164	1	0.181	0.164	2	0.000	0.000
16	3	0.004	0.002	-	-	-	-	-	-	1	0.133	0.142	2	0.000	0.000
17	1	0.104	0.091	-	-	-	-	-	-	2	0.016	0.019	1	0.000	0.000
18	1	0.097	0.072	-	-	-	-	-	-	1	0.097	0.072	-	-	-
19	1	0.058	0.073	-	-	-	-	-	-	-	-	-	1	0.000	0.000
20	1	0.041	0.040	1	0.041	0.040	-	-	-	-	-	-			
21	-	-	-	-	-	-	1	0.026	0.038	-	-	-	1	0.000	0.000
28	-	-	-	1	0.001	0.000	-	-	-	-	-	-	-	-	-

**Table 7:** ALL (9 subtypes) - Significance comparison of size of subnetworks induced by high-variance genes in preprocessed output; *p* = *p*-value of the frequency using first three principal components

	Raw		Scaled		Z-score		Quantile		GFS	
Size	freq	*p*	freq	*p*	freq	*p*	freq	*p*	freq	*p*
2	87	0.672	77	0.861	76	0.876	87	0.672	80	0.071
3	44	0.621	46	0.545	41	0.722	45	0.577	67	0.000
4	24	0.483	24	0.483	24	0.483	23	0.546	39	0.000
5	18	0.105	18	0.105	18	0.105	18	0.105	16	0.001
6	3	0.890	2	0.958	4	0.804	2	0.958	11	0.000
7	9	0.025	4	0.408	3	0.588	9	0.025	4	0.029
8	2	0.492	3	0.289	4	0.144	3	0.289	4	0.013
9	5	0.017	6	0.004	4	0.057	5	0.017	1	0.170
10	3	0.062	3	0.062	4	0.021	2	0.165	5	0.000
11	3	0.038	7	0.001	7	0.001	3	0.038	2	0.015
12	1	0.289	3	0.021	2	0.092	2	0.092	3	0.001
13	1	0.230	2	0.059	4	0.007	-	-	1	0.011
14	3	0.005	1	0.203	-	-	3	0.005	2	0.000
15	1	0.193	1	0.193	1	0.193	2	0.047	2	0.002
16	1	0.124	1	0.124	2	0.031	1	0.124	2	0.000
17	1	0.122	1	0.122	-	-	1	0.122	-	-
19	-	-	-	-	-	-	-	-	1	0.000
20	-	-	-	-	-	-	-	-	1	0.000
23	1	0.006	-	-	-	-	1	0.006	-	-
24	-	-	1	0.003	1	0.003	-	-	-	-

### Effect of sample size on performance of GFS

**Fig. 10:**
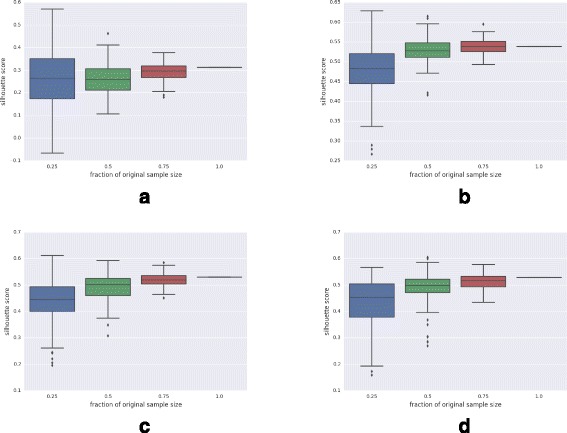
Effect of sample size on clustering performance of GFS. **a** ALL (9 Subtypes). **b** ALL (2 Subtypes). **c** DMD. **d** Leukemia

To examine the effect of sample size on GFS, we randomly selected samples of the size of 0.25, 0.50, 0.75 times the original sample size over 100 iterations. We then noted the range of silhouette scores obtained from the iterations for each sample size. (For the heterogeneous ALL dataset, the first three PCs were used to calculate the silhouette scores, while for the other datasets, only the second and third PCs were used). As expected, Fig. 10 shows that the clustering performance improves with increase in sample size. Interestingly, the boxplots in Fig. 10, interpreted together with Tables 2 and 3, also indicate that the median performance of GFS when provided with even 0.25 times of the entire sample size is still comparable with, and often better than, that of other normalization methods when they are supplied with the entire sample size.

## Conclusion

An effective preprocessing technique is expected to transform the gene expression matrix such that data of the same phenotype from different sources is made similar. This can be achieved by removing or accounting for obscuring noise in gene expression measurement, and retaining interesting variation relevant to properties of biological interest. Such a processing is essential to ensure reliable downstream analysis of gene expression data. However, popular normalization techniques do not necessarily improve the quality of expression data, and sometimes even exacerbate the issue by mistaking real variation for noise and discarding it.

We discussed a new approach, Gene Fuzzy Score, to address this issue and compared it with other popular preprocessing methods with respect to three important criteria. First, we assessed the capability of the transformed output of each technique to resolve differences in phenotypes within the dataset. Secondly, we estimated the consistency of their output when presented with different datasets with the same phenotype distribution. Finally, we analysed the distributions of size of subnetworks induced by genes indicated to be sources of interesting variation in each processed expression matrix. In each of these aspects, GFS was successful in improving the transformation outcome, proving its applicability in datasets with batch effects and heterogeneity. Moreover, the performance of GFS improves with increase in sample size.

A recurring observation from our experiments is that in datasets with significant batch effects, the batch effects are generally captured by the first principal component in PCA. Thus, applying a PCA transformation and excluding the first principal component from subsequent analysis leads to significant reduction in batch effects in any dataset, and improves the performance of all preprocessing techniques. Further, we note that GFS outperforms other methods irrespective of whether this additional step is implemented.

Another merit of GFS is the interpretability of its transformed outcome. A biologist may quickly understand how highly the gene is ranked in a particular patient. For b, when a gene has a GFS score of 0.5 in a patient, it means the gene is in the top 10% most highly expressed genes in that patient (assuming *θ*_1_and *θ*_2_ are set at 5 and 15*%* respectively). Thus, apart from being a robust and effective preprocessing technique, GFS is also easily interpretable.

While we evaluated GFS only on microarray gene expression, it is conceivable that the method may be applied to data obtained from other high-throughout technologies such as RNA-seq and SWATH proteomics. Exploring this possibility remains the subject of our future work.

## References

[1] Shi L, Reid LH, Jones WD, Shippy R, Warrington JA, Baker SC (2006). The MicroArray Quality Control (MAQC) project shows inter-and intraplatform reproducibility of gene expression measurements. Nat Biotechnol.

[2] Luo J, Schumacher M, Scherer A, Sanoudou D, Megherbi D, Davison T (2010). A comparison of batch effect removal methods for enhancement of prediction performance using MAQC-II microarray gene expression data. Pharmacogenomics J.

[3] Leek JT, Scharpf RB, Bravo HC, Simcha D, Langmead B, Johnson WE (2010). Tackling the widespread and critical impact of batch effects in high-throughput data. Nat Rev Genet.

[4] Raser JM, O’Shea EK (2005). Noise in gene expression: origins, consequences, and control. Science.

[5] Cheadle C, Vawter MP, Freed WJ, Becker KG (2003). Analysis of microarray data using Z-score transformation. J Mol Diagn.

[6] Goh WWB, Guo T, Aebersold R, Wong L (2015). Quantitative proteomics signature profiling based on network contextualization. Biol Direct.

[7] Lim K, Wong L (2014). Finding consistent disease subnetworks using PFSNet. Bioinformatics.

[8] Geistlinger L, Csaba G, Küffner R, Mulder N, Zimmer R (2011). From sets to graphs: towards a realistic enrichment analysis of transcriptomic systems. Bioinformatics.

[9] Goh WWB, Wong L (2016). Evaluating feature-selection stability in next-generation proteomics. J Bioinforma Comput Biol.

[10] Haslett JN, Sanoudou D, Kho AT, Bennett RR, Greenberg SA, Kohane IS (2002). Gene expression comparison of biopsies from Duchenne muscular dystrophy (DMD) and normal skeletal muscle. Proc Natl Acad Sci USA.

[11] Pescatori M, Broccolini A, Minetti C, Bertini E, Bruno C, D’amico A (2007). Gene expression profiling in the early phases of DMD: a constant molecular signature characterizes DMD muscle from early postnatal life throughout disease progression. FASEB J.

[12] Armstrong SA, Staunton JE, Silverman LB, Pieters R, den Boer ML, Minden MD (2002). MLL translocations specify a distinct gene expression profile that distinguishes a unique leukemia. Nat Genet.

[13] Golub TR, Slonim DK, Tamayo P, Huard C, Gaasenbeek M, Mesirov JP (1999). Molecular classification of cancer: class discovery and class prediction by gene expression monitoring. Science.

[14] Yeoh EJ, Ross ME, Shurtleff SA, Williams WK, Patel D, Mahfouz R (2002). Classification, subtype discovery, and prediction of outcome in pediatric acute lymphoblastic leukemia by gene expression profiling. Cancer Cell.

[15] Ross ME, Mahfouz R, Onciu M, Liu HC, Zhou X, Song G (2004). Gene expression profiling of pediatric acute myelogenous leukemia. Blood.

[16] Rousseeuw PJ (1987). Silhouettes: a graphical aid to the interpretation and validation of cluster analysis. J Comput Appl Math.

[17] Soh D, Dong D, Guo Y, Wong L (2010). Consistency, comprehensiveness, and compatibility of pathway databases. BMC Bioinforma.

